# Use of Ceftazidime-Avibactam in Children Admitted to Pediatric Intensive Care Units

**DOI:** 10.3390/children11060664

**Published:** 2024-05-29

**Authors:** André Ricardo Araujo da Silva, Rafael Quijada

**Affiliations:** 1 Faculty of Medicine, Universidade Federal Fluminense, Niterói 24220-900, Brazil; rafaelquijada@id.uff.br; 2 Prontobaby Group, Rio de Janeiro 20540-100, Brazil

**Keywords:** pediatric intensive care unit, ceftazidime-avibactam, treatment

## Abstract

Background: Ceftazidime-Avibactam (CAZ-AVI) is one of the new antibiotics available to treat infections due to carbapenem-resistant gram-negative bacteria (CRB). Our aim was to describe the use of CAZ-AVI in children admitted to pediatric intensive care units (PICUs), with suspected or proven CRB infections. Methods: A retrospective descriptive study was conducted in two PICUs of Rio de Janeiro, Brazil, between January 2020 and January 2024. Children aged 0 to 18 years who received CAZ-AVI for more than 24 h were included. Results: CAZ-AVI was used in 37 patients. The median age was 28 months (range 1–215), 17 (45.9%) being male. The median time from the patient admission to the initial prescription of CAZ-AVI was 39.9 days (range 1–138). Thirty-four (91.9%) children had at least one comorbidity at admission and (91.9%) used at least one invasive device prior to the CAZ-AVI prescription, and 89.2% had received carbapenem before; and fifteen (40.5%) had healthcare-associated infection (HAI) prior to CAZ-AVI use. The mean time of CAZ-AVI use was 11 days (range 1–22). Gram-negative bacteria were isolated in cultures from 12 (32.4%) patients in the 24 h prior to prescription or on the day of prescription. In five patients, CRB was confirmed in cultures, and in four (80%) of them, microbiological clearance was verified after 7 days of treatment. The 30-day mortality rate was 37.8%. Conclusion: Almost all patients who used CAZ-AVI were critically ill children with multiple comorbidities and previous use of carbapenems. Among CRB confirmed infections, microbiology clearance in 7 days was high.

## 1. Introduction

In 2017, the World Health Organization (WHO) published a list of antibiotic-resistant priority pathogens. The list particularly highlights the threat of multidrug-resistant gram-negative bacteria, emphasizing the need for research and development of new antibiotics to address the challenge of resistance [1]. The critical pathogens cited by WHO as priority 1 include *Acinetobacter baumannii* and *Pseudomonas aeruginosa* which are carbapenem-resistant and *Enterobacteriaceae* which is carbapenem-resistant and ESBL-producing.

At least five new drugs have addressed multidrug resistant infections in the last few years, including children in trials [ceftazidime-avibactam (CAZ-AVI), ceftolozane-tazobactam, imipenem-relebactam, meropenem-vaborbactam, and cefiderocol] [2]. Usually, the research on the development of new antibiotics in children, particularly in neonates, is conducted shortly after an adult trial. Considering that this population is also affected by resistance, two additional antibiotics are potential candidates for accelerated development (cefepime + taniborbactam and sulbactam + durlobactam) [3].

CAZ-AVI is a new antibiotic against gram-negative infections used in many countries worldwide and approved for children in Brazil, for complicated intra-abdominal infections (in association with metronidazole) and complicated urinary tract infections. In adults, indications included hospital ventilator-associated pneumonia and bacteremia associated with one of the above indications.

The safety profile for complicated intra-abdominal infection (cIAI) in children ≥3 months to <18 Years with complicated urinary tract infection was evaluated by Bradley et al. in 68 children, between September 2i015 and September 2017, in nine countries. Similar rates of adverse effects were found in CAZ-AVI and control groups, and favorable clinical and microbiologic responses were reported in over 90% of patients [4]. In a case series of eight patients (neonates and children < 5 years) with suspected/proven infections due to extensively drug-resistant (XDR) or pan-drug resistant (PDR) *Klebsiella pneumoniae* treated with CAZ-AVI, there were clinical and microbiological responses, with no treatment discontinuation due to adverse events and no deaths after 30 days [5]. Considering the need for CAZ-AVI evaluation of in clinical practice, this study aims to describe the profiles of children who used CAZ-AVI in pediatric intensive care units (PICUs), with suspected or proven infections due to gram-negative bacteria resistant to carbapenems.

## 2. Materials and Methods

A retrospective descriptive study was conducted in two pediatric intensive care units. The units are located in the city of Rio de Janeiro, Brazil, in two different hospitals. Both units received clinical and surgical patients from the emergency room, the pediatric wards, and transferred from other hospitals. Units 1 and 2 have 10-bed and 15-bed capacities, respectively, and a 90% occupancy rate for both PICUs. We included children between 0 and 18 years old who used CAZ-AVI for more than 24 h between January 2020 and January 2024. The indication for CAZ-AVI was complicated intra-abdominal infection, complicated urinary tract infection, bacteremia, sepsis, or respiratory tract infection due to suspected or proven gram-negative resistant to carbapenems. The decision to initiate CAZ-AVI was a consensus between the chief physician of the PICU and the pediatric infectious disease specialist, based on the high likelihood of infection with carbapenem-resistant bacteria (CRB). Patients transferred or dead within 24 h after admission were excluded.

Both hospitals implanted a program of antimicrobial stewardship in November 2016, and one of the key points was the restriction of selected antimicrobials and their prescription only after consultation with pediatric infectious disease specialists. CAZ-AVI was included in the list of restricted antimicrobials in 2019, after approval for prescription by the Brazilian National Agency of Surveillance in children. When a restricted antimicrobial is needed for a child in the studied institutions in any ward or intensive care unit, the case is first discussed prior with the pediatric infectious specialist. After the agreement of the specialist, the antimicrobial is prescribed in the local electronic system for review of dose, interval, and duration by the pharmacist. If any errors are detected, the prescription is denied, and a correction is made.

The following doses were used in children between 3 months and 5 months: 120 mg ceftazidime/kg/day q8h infused over 2 h, and in children > 6 months: 150 mg ceftazidime/kg/day q8h infused over 2 h, maximum 6 g/day.

Gram-negative bacteria accounted for 66/129 (51.2%) and 70/176 (39.8%) of all HAI in hospitals 1 and 2, respectively, between 2016 and 2022. CRB was the cause of 5/129 (3.9%) of healthcare-associated infections (HAI) in hospital 1, between 2016 and 2022, and 3/176 (1.7%) in hospital 2 during the same period.

We analyzed the duration of CAZ-AVI, and possible risk factors for acquiring CRB, such as previous use of an invasive device, healthcare-associated infections, surgery, parenteral nutrition and carbapenem use, length of stay, and 30-day outcomes (discharge, still admitted, or death). Children were analyzed in two subgroups (survivors and non-survivors after 30 days of CAZ-AVI prescription). The safety of CAZ-AVI was not analyzed in this manuscript. The Pediatric Index of Mortality (PIM 2) was calculated for all patients at admission. The data were compiled in an Excel file. A descriptive analysis and means, medians, the Mann–Whitney test for comparison of continuous variables, and Fisher’s exact test for categorical variables were used. A *p*-value less than 0.05 was considered statistically significant.

## 3. Results

CAZ-AVI was prescribed for 37 patients, with 2 prescriptions in 2020, 17 in 2021, 9 in 2022, 9 in 2023, and 0 in 2024. The median age was 28 months (range 1–215), 17/37 (45.9%) were male, and 34/37 (91.9%) had pre-existing chronic disease before the current admission. Five out of thirty-seven patients (13.5%) had COVID-19 before the prescription of CAZ-AVI, one in 2020, two in 2021 and two in 2022. Twenty-eight patients were evaluated with PIM 2 at admission between 0 and 10%, and in nine, the score was higher than 10%. Twenty-two patients received 150 mg/kg/day q8h infused over 2 h, maximum 6 g/day, eight patients received 6 g (ceftazidime)/day (2 g/ceftazidime 8/8 h) infused over 2 h, and seven received 120 mg/kg/day q8h infused over 2 h. All doses were adequate according to the child’s weight.

The main reasons for prescription were the following: lower respiratory tract infection in 17/37 (45.9%), sepsis or septic shock in 6/37 (16.2%), two or more sites of infection simultaneously in 4/37 (10.8%), gastrointestinal tract infection in 2/37 (5.4%), central nervous system infection in 2/37 (5.4%), gastrointestinal infection in 2/37 (5.4%), and other reasons in 4/37 (10.8%) patients (blood culture positive, skin infection, meropenem allergy, and upper respiratory tract infection). The 30-day mortality rate was 37.8% (14/37). All 37 patients studied received a different antibiotic before the prescription of CAZ-AVI. Table 1 shows the previous data of patients in the period in the PICUs, before the prescription of CAZ-AVI in survivors and non-survivors.

The median duration of prior mechanical ventilation was 17 days (1–138), 22.2 days (0–138) for the central venous catheter, and 3 days (0–43) for the urinary tract catheter. The median duration of previous parenteral nutrition was 0 days (0–89). Hemodialysis was required in two patients (both survivors), and neither required an extracorporal circulation system.

An infectious agent was isolated in 16 (43.2%) patients, and 12 (32.4%) of them were gram-negative bacteria patients at the time of CAZ-AVI prescription. Among patients with gram-negative samples, mortality was 5/12 (41.6%). In five patients, CRB was confirmed in cultures, and in four (80%) of them, microbiological clearance was verified after 7 days of treatment. Considering the 30-day outcomes, one patient was discharged, one remained hospitalized, and three died. The profiles of these patients are shown in Table 2.

In the remaining 21 patients where no bacteria or fungi were isolated, no infectious agent was found; 1 patient was discharge; 13 were still admitted; and 7 had died. Mortality in cases confirmed with CRB was 3/5 (60%) and 7/21 (33.3%) in patients without an isolated infectious agent (*p* = 0.19).

The median time of prior antibiotic use in children with CRB-confirmed infections was 6 days and 29 days in children without agent isolated in cultures (*p* = 0.030).

The agents and site of infection are shown in Table 3.

The median length of stay until the prescription of CAZ-AVI was 39.9 days (1–138) and the median time of treatment was 11 days (1–22). The median time of treatment was 14 days in the survival group × 6.5 days in the non-survival group (*p* < 0.0001).

## 4. Discussion

The global crisis of antibiotic resistance also affects children admitted to pediatric intensive care units, where broad-spectrum treatment with antibiotics is usually necessary to achieve favorable outcomes. In a multicenter study in four PICUs in Greece, the rates of resistance of carbapenem *Pseudomonas aeruginosa* and *Acinetobacter baumannii* samples related to healthcare-associated infections were 44% and 80%, respectively [6]. In our study, our historical series showed a percentage of less than 10% of carbapenem-resistant bacteria causing HAI in PICUs, in both units.

Recent guidance from the Infectious Diseases Society of America recommends CAZ-AVI as an option for treating target organisms such as carbapenem-resistant Enterobacterales and Pseudomonas aeruginosa with difficult-to-treat resistance (DTR-*P. aeruginosa*) in adults and children [7]. Unlike in adults, few new options are available to treat these organisms in Brazil, and CAZ-AVI represents an option for critically ill children admitted to PICUs

CAZ-AVI has been used in our institution only following strict criteria and after discussion with pediatric infectious disease specialists (PIDS) for selected patients with critical diseases and a risk of death. The local politics of the antimicrobial stewardship program are very strict, and the prescription of CAZ-AVI and other restricted antimicrobials is not allowed without pre-authorization, even during nights, weekends, and holidays. The only exceptions for prescription of restricted antimicrobials without pre-authorization are patients with sepsis and febrile neutropenia, when restricted antimicrobials could be administered within the first hour after diagnosis, but subsequent doses require PIDS authorization. At the present moment, CAZ-AVI represents the last option for treating suspected CRB infections in both studied PICUs, and its introduction into clinical practice was possible after discussion with local specialists and Brazilian approval for pediatric use.

A recent report described the use of CAZ-AVI in 38 children admitted to a single center in China between 2022 and 2022 [8], but the authors included children also admitted to other wards than the PICUs. Unlike our study, where all children were in PICUs, only 34.2% of the Meng’s study patients were in intensive care units at the time of CAZ-AVI treatment.

Most of the patients who received CAZ-AVI were admitted in 2021, during the second year of the COVID-19 pandemic. Despite this finding, our small number of patients, especially those admitted with COVID-19, does not allow us to infer that this result could be attributed to the COVID-19 pandemic, considering that the vaccine was not available during that period, and we could not evaluate the gravity score at admission. The effects of COVID-19 pandemics on the increased prevalence of multidrug-resistant bacteria in ICUs causing HAI are uncertain, but in hospitals without infection prevention and control and/or antimicrobial stewardship program initiatives, an increase in gram-negative AMR was verified in a systematic review and meta-analysis (risk ratio 1.11, 95% CI: 1.03–1.20) [9].

Despite a low PIM 2 score at admission, almost all patients remained in the PICU for many days until the CAZ-AVI prescription and were exposed to well-described risk factors for possible CRB acquisition during their stay. Almost all patients had previous illness, a longer stay of hospitalization before CAZ-AVI, and multiple and long-term use of invasive devices such as mechanical ventilation, central venous catheter, and urinary tract infections. Liu and colleagues, in a meta-analysis, reported sixteen risk factors for carbapenem-resistant *Klebsiella pneumoniae* infections, with longer hospital stay (OR = 12.92) and more days of ICU stay (OR = 4.58) being the most important [10].

The 30-day mortality of all patients was higher (37.8%) and also showed elevated values when only patients with gram-negative isolates were analyzed (41.6%). Iosifidis and colleagues did not report deaths in neonates and children <5 years treated with CAZ-AVI for extensively drug-resistant (XDR) or pan drug-resistant (PDR) *Klebsiella pneumoniae* infections, but only eight children were included in the single-center case series [5]. Considering the few reports in children about mortality from carbapenem-resistant bacteria, more data are available from the adult population. In a systematic review and meta-analysis of mortality in adult patients infected with carbapenem-resistant *Klebsiella pneumoniae* from North America, South America, Europe, and Asia, the values were 33.24, 46.71, 50.06, and 44.82%, respectively [11]. Usually, mortality in PICUs ranges from 2.31% to 25%, but is higher in patients with critical conditions like septic shock [12,13,14]. Considering that our children had several risk factors for unfavorable outcomes, the mortality rate is compatible with the patients’ diseases.

The low number of patients with confirmed CRB prevents a specific analysis of efficacy in this subpopulation. When considering microbiological clearance, in this subgroup, microbiological clearance was high, but on the other hand, only one received discharge.

Until 2023, CRB accounted for less than 4% of all HAI in both PICUs studied, despite the units being references for the treatment of critically ill children in the city of Rio de Janeiro. Our efforts are ongoing, with an active surveillance of children colonized with CRB and measures to prevent the spread of these agents in hospitals contributing to maintaining low rates of CRB causing HAIs, which are considered for the studied hospitals as a local quality marker of care. The prevalence of CRB-causing infection in children varies according to the ward, type of gram-negative bacteria, and type of associated infection, being high in neonatal intensive care units and in pediatric oncologic wards [15].

Our study has some limitations. Firstly, it included patients with suspected CRB infections, but even when confirmed infections were analyzed, mortality was almost the same for the whole group. In addition, the existence of a robust local antimicrobial stewardship allowed the prescription of CAZ-AVI only when a strong suspicion of CRB was possible. Secondly, the inclusion of two centers for critically ill children, while previous reports included fewer patients than ours and only single centers or case reports [16,17]. The third limitation is the absence of CAZ-AVI safety in the children studied, and the last limitation was the study design (retrospective study), in which it was not possible to control the quality of the information. However, considering that the data were entered into electronic medical records, it is unlikely that there would be incorrect data for analysis. To our knowledge, this is the largest Brazilian series reporting CAZ-AVI treatments in children.

## 5. Conclusions

Almost all patients who used CAZ-AVI were critically ill children, with multiple comorbidities and previous use of carbapenems. Among the confirmed CRB infections, microbiological clearance at 7 days was high.

## Figures and Tables

**Table 1:** Previous data before prescription of CAZ-AVI, including possible risk factors for carbapenem-resistant bacteria acquisition (Rio de Janeiro, city 2020–2024).

Variables	Survivors N = 23	Non-Survivors N = 14	*p* Value #
Length of stay in PICU until CAZ-AVI prescription (median in days) Previous surgery (%)	31 60.9	21.5 42.9	0.52 * 0.33
Previous healthcare-associated infection (%)	39.1	42.9	1.0
Previous use of invasive device (%) Previous use of mechanical ventilation Previous use of central venous catheter Previous use of urinary tract infection	78.3 82.6 52.2	78.6 100 57.1	1.0 0.28 1.0
Previous use of parenteral nutrition (%)	21.7	28.6	0.70
Colonization by multidrug resistant bacteria (%)	60.9	57.1	1.0
Prior use of antibiotic (%)	100	100	1.0
Previous use of third/fourth generation cephalosporin Previous use of carbapenems	52.2 82.6	57.1 100	1.0 0.28
Median of time of antibiotic use before CAZ-AVI prescription (in days)	39	20.5	0.18 *

* Mann–Whtiney test # Fisher’s exact.

**Table 2:** Profile of CRB confirmed cases (Rio de Janeiro, city 2020–2024).

	Age (in Months)	Gender	Type of Prior Comorbidity	Prior CRB Colonization	Previous Use of Carbapenems	CAZ-AVI Dosage	Infection Site at CAZ-AVI Admission
Patient 1	215	Female	NPE	Yes	No	2 g 8/8 h infused over 2 h	Pneumonia
Patient 2	30	Male	NPE	No	No	120 mg/kg/day q8h infused over 2 h	Pneumonia
Patient 3	17	Female	Central nervous system tumor	Yes	Yes	150 mg/kg/day q8h infused over 2 h	Urinary tract infection
Patient 4	172	Female	NPE/Hydrocephalus	No	Yes	2 g 8/8 h q8h infused over 2 h	Pneumonia/urinary tract infection/Central nervous system
Patient 5	96	Male	Arthrogryposis	Yes	Yes	150 mg/kg/day q8h infused over 2 h	Bloodstream infection

CRB: Carbapenem-resistant bacteria, CAZ-AVI: Ceftazidime-avibactam, NPE: Non-progressive encephalopathy.

**Table 3:** Agents isolated and sites in children with CAZ-AVI prescription (Rio de Janeiro, city 2020–2024).

Agents	Blood	Urine	Tracheal Aspirate	Sputum	Surgical Site Aspirate	Cerebrospinal Fluid	Total
*Escherichia coli*	0	1	0	0	0	0	1
*Klebsiella pneumoniae*	1	0	0	0	0	0	1
*Pseudomonas aeruginosa*	1	1	1	0	0	0	3
**Carbapenem-resistant** ***Acinetobacter baumannii***	0	0	1	0	0	0	1
**Carbapenem-resistant** ***Pseudomonas aeruginosa***	0	1	2	0	0	0	3
*Escherichia coli* ESBL	0	0	0	0	1	1	2
**Pan-resistant** ***Klebsiella pneumoniae***	0	0	0	0	0	1	1
*Staphylococcus aureus*	0	0	0	1	0	0	1
*Sacaromyces cerivisiae*	0	0	0	1	0	0	1
*Candida* sp.	0	2	0	0	0	0	2
Total	2	5	4	2	1	2	16

## Data Availability

The data presented in this study are available on request from the corresponding author. The data are not publicly available due to privacy, legal and ethical reasons.
